# *Helicobacter pylori *with stronger intensity of CagA phosphorylation lead to an increased risk of gastric intestinal metaplasia and cancer

**DOI:** 10.1186/1471-2180-11-121

**Published:** 2011-05-27

**Authors:** Chiao-Hsiung Chuang, Hsiao-Bai Yang, Shew-Meei Sheu, Kuei-Hsiang Hung, Jiunn-Jong Wu, Hsiu-Chi Cheng, Wei-Lun Chang, Bor-Shyang Sheu

**Affiliations:** 1 Department of Internal Medicine, Medical College, National Cheng Kung University, Sheng-Li Road, Tainan, Taiwan; 2 Department of Pathology, Medical College, National Cheng Kung University, Sheng-Li Road, Tainan, Taiwan; 3 Institute of Basic Medical Sciences, Medical College, National Cheng Kung University, Sheng-Li Road, Tainan, Taiwan; 4 Institute of Clinical Medicine, Medical College, National Cheng Kung University, Sheng-Li Road, Tainan, Taiwan

**Keywords:** *H. pylori*, *cagA*, CagA phosphorylation, intestinal metaplasia, gastric cancer

## Abstract

**Background:**

Nearly all Taiwanese *H. pylori *stains are *cagA*-genopositive and encode CagA protein. In this study, we evaluated whether different intensity of tyrosine phosphorylated-CagA (p-CagA) had an impact on the clinical diseases and histological outcomes in this area.

**Results:**

We enrolled 469 dyspeptic patients and prospectively obtained the gastric biopsy specimens and the *H. pylori *isolates. These patients were categorized according to the clinical diseases, such as duodenal ulcer, gastric ulcer, gastric cancer, and gastritis with or without intestinal metaplasia. Their gastric specimens were reviewed by the updated Sydney's system. Furthermore, a total of 146 patients were randomly selected from each clinical category for evaluation of their isolates' p-CagA intensity by *in vitro *AGS cells co-culture. The p-CagA was sparse in 30 (20.5%), weak in 59 (40.5%), and strong in 57 (39%) isolates. The isolates from the patients of gastric cancer or gastritis with intestinal metaplasia had stronger p-CagA intensity than those of gastritis without intestinal metaplasia (*p *≤ 0.002). Moreover, the patients infected with isolates with strong or weak p-CagA intensity had a higher risk of gastric intestinal metaplasia (*p *< 0.05, odds ratio 3.09~15.26) than those infected with sparse p-CagA isolates.

**Conclusions:**

Infection with *H. pylori *stains with stronger p-CagA intensity may lead to an increased risk of gastric intestinal metaplasia and cancer.

## Background

The *cagA *gene encoded CagA protein is a well-known virulent factor of *Helicobacter pylori*, which is associated with an increased risk of peptic ulcer or even gastric cancer [1-4]. The CagA protein can be tyrosine phosphorylated in the gastric epithelial cells via the type IV secretion system translocation [5]. The phosphorylated-CagA (p-CagA) mediates interleukin-8 secretion, enhances gastric inflammation, and clinical diseases [5-8]. As shown in the Mongolian gerbil models, *H. pylori *isolates with functional type IV secretion system could induce more CagA phosphorylation and severer gastric inflammation and intestinal metaplasia (IM) [9,10]. However, there is no adequate clinical evidence in a setting to support the relationship between CagA phosphorylation intensity and the risk of gastric carcinogenesis.

In the western countries, about 70% or less of clinical *H. pylori *strains are *cagA-*genopositive [11,12]. In contrast, in the eastern countries, such as in Taiwan, there is a nearly 100% prevalence of *cagA-vacA-babA2 *triple-positive *H. pylori *strains [13-15]. Moreover, most strains in East-Asia, and also Taiwan, encoded CagA contain EPIYA-ABD motif [16-18]. Our previous data supported 100% positive of some genes which are encoded from *cag *pathogenicity island (PAI), such as *cagC, cagE, cagF, cagN, and cagT *[19]. Accordingly, because of the universal presence of genes in *cag*-PAI in Taiwan, this region should be suitable to answer whether different p-CagA intensity are related to different clinicopathologic outcomes of *H. pylori *infections. The study is highly original to illustrate the p-CagA intensity could be diverse among the *cagA*-positive *H. pylori *isolates, and to support *H. pylori *with stronger p-CagA intensity can increase the risk of gastric carcinogenesis.

## Methods

### Patients and study design

Patients with recurrent dyspepsia symptoms, who received upper gastrointestinal endoscopy, were consecutively enrolled, once they were proven to have a *H. pylori *infection defined by a positive result of culture. None of them had a previous history of anti-*H. pylori *therapy. For each patient, the gastric biopsies were obtained during the endoscopy for *H. pylori *culture and histological analysis. This study were approved by 'Human Experiment and Ethics Committee of National Cheng Kung University Hospital' (ER-97-245) and all the patients signed the informed consents before enrollment.

A total of 469 patients (264 women and 205 men; mean age 48.1 years) were enrolled, including 26 with gastric cancer, 64 with gastric ulcer, 131 with duodenal ulcer, 209 with gastritis & without IM and 39 with gastritis & IM. From each category, 32 isolates were randomly sampled (the cancer group had just 26 isolates and all were selected). A total of 154 isolates were sampled, but 8 stored strains could not be successfully subcultured after refrigeration. Accordingly, 146 strains were finally obtained from patients with duodenal ulcer (n = 31), gastric ulcer (n = 32), gastric cancer (n = 24), gastritis with IM (n = 28), and gastritis without IM (n = 31). These 146 *H. pylori *isolates were analyzed for the *cagA*-genotype by polymerase chain reaction and for the intensity of p-CagA by *in vitro *co-culture with AGS cells (a human gastric adenocarcinoma epithelial cell line); further the p-CagA intensity was defined as strong, weak, or sparse. Besides, in each patient, their gastric biopsies taken from both antrum and corpus for histology were reviewed by the updated Sydney's system.

### Histological analysis of the gastric specimens

Each gastric sample was stained with haematoxylin and eosin as well as with modified Giemsa stains to analyze for *H. pylori *density (HPD, range 0-5) and *H. pylori-*related histology by the updated Sydney's system. The histological parameters included acute inflammation score (AIS, range 0-3; 0: none, 1: mild, 2:moderate, 3: severe), chronic inflammation score (CIS, range 1-3; 1: mild, 2: moderate, 3: severe), mucosal atrophy, and IM as applied in our previous studies [20,21]. For each patient, the presence of atrophy or IM was defined as a positive histological finding in any specimen from the antrum or corpus. In each patient, the total HPD, AIS, and CIS were the sum of each score of the gastric specimens from antrum and corpus, and thus ranged from 0-10, 0-6, and 2-6, respectively. Based on the sum of HPD, the patients were categorized as loose (score ≤ 5), moderate (score within 6-8), and dense (score ≥ 9) *H. pylori *colonization, respectively. For the sum of AIS, mild, moderate, and severe acute inflammations were defined with scores ≤1, 2-3, or ≥4, respectively. Based on the sum of CIS, mild, moderate, and severe chronic inflammations were defined with scores ≤3, 4-5, or 6, respectively.

Based on the specimens collected from both the antrum and corpus within the same patient, the topographical distribution of chronic gastritis was defined as follows: 1) very limited chronic gastritis, if the CIS scored was 1 for both antrum and corpus; 2) antrum-predominant gastritis, if the CIS score of the antrum was higher than the score of the corpus; and 3) corpus-predominant gastritis, if the corpus CIS was equal to or higher than that of the antrum [21].

### Analysis of cagA genotype and type IV secretion system function of H. pylori

**Figure 1:**
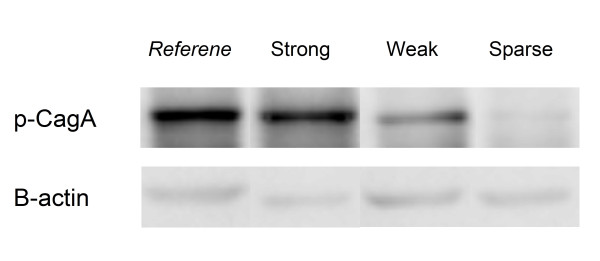
**The p-CagA and β-actin immunoblot gel imaging of the reference strains (Hp830) and the representative strain in each subgroup**.

All *H. pylori *isolates were re-sorted by culture as applied in our previous publications [14,15]. The genomic DNA of these collected isolates was then extracted for polymerase chain reaction to verify the *cagA-*genotype by primers used in our published article [19]. To analyze the p-CagA intensity of each strain, *H. pylori *strains (2 × 10^8 ^cells) were suspended in 0.5 mL of phosphate-buffered saline (PBS) and were co-cultured with 2 × 10^6 ^AGS cells at a multiplicity of infection (MOI) of 100 for 5 hours. Afterward, the culture medium was removed and the AGS cells were lysed after five times washing with PBS. The AGS lysates were applied to SDS-PAGE gel electorphoresis and transferred to membranes for western blots analysis. A phosphorylated tyrosine antibody and anti-actin antibody (Santa Cruz Biotechnology, Inc, Santa Cruz, CA) were used to detect the p-CagA and β-actin proteins. A clinical *H. pylori *strain (Hp830) which had a strong p-CagA band in the western blots was used as reference. In each western blots procedure, 7-9 clinical strains and the reference strain were analyzed in the same run. The relative immunoblot density of the p-CagA and β-actin proteins were quantitated by scanning the images on a gel analysis system (BioSpectrum AC Imaging System, Vision Work LS software, Upland, CA) for each strain and defined as [p-CagA] and [Bactin]. The amount of p-CagA and β-actin proteins of the reference strain in the same run were also semi-quantified as reference and defined as [p-CagA-ref] and [Bactin-ref]. The p-CagA intensity of each strain was calculated by the formula: *p-CagA value = ([p-CagA]/[Bactin])/([p-CagA-ref]/[Bactin-ref])*. Strains with a p-CagA value <0.2, 0.2-0.8, and >0.8 were defined as sparse, weak, and strong p-CagA intensity. The immunoblot gel imaging of the representative strain in each subgroup and the reference strain (Hp830) were showed in Figure 1.

### Statistical analysis

SPSS software version 12.0 for Windows (SPSS Inc., Chicago, IL) was used for the statistical analysis. The differences in the p-CagA intensity among the subgroups of patients were analyzed by Pearson chi-square test. The odds ratio on the risk of IM and corpus-predominant gastritis between the different subgroups were analyzed by the logistical regression. All tests were two-tailed, and a p value less than 0.05 were considered significant.

## Results

### H. pylori isolates with diverse p-CagA intensity

From the 469 patients, we sampled 146 strains for the analysis of the p-CagA intensity. The clinical characteristics of these patients were shown in Table 1. In each sampled group, age and gender were matched between the sampled patients and the entire group of patients (p = NS). All of the 146 enrolled *H. pylori *isolates were *cagA*-genopositive and the p-CagA intensity was sparse in 30 (20.5%), weak in 59 (40.5%), and strong in 57 (39%) isolates.

**Table 1:** The clinical characteristics between all patients with isolated *H. pylori *strains and the selected patients for analysis of the p-CagA intensity of the strains

	Patients with *H. pylori *cultures (n = 469)	Selected patients for p-CagA analysis (n = 146)	*p *value*
Age (year [mean ± SD])	48.1 ± 14.2	50.4 ± 16.3	NS
Gender (F/M)	264/205	73/73	NS
Endoscopic diagnosis (year; n(F/M))			
Gastritis			
- without intestinal metaplasia	44.3; 209 (137/72)	41.2; 31 (18/13)	NS
- with intestinal metaplasia	54.5; 39 (29/10)	57.0; 28 (22/6)	NS
Duodenal ulcer	48.0; 131 (68/63)	46.6; 31 (14/17)	NS
Gastric ulcer	51.3; 64 (17/47)	49.5; 32 (7/25)	NS
Gastric cancer	60.4; 26 (13/13)	60.6; 24 (12/12)	NS

* Either the age or the gender was matched between the 146 selected patients and the entire patients in each sampled groups (Pearson chi-square test for gender & Student's *t *test for age analysis).

### Stronger p-CagA intensity may lead to intestinal metaplasia & gastric cancer

**Figure 2:**
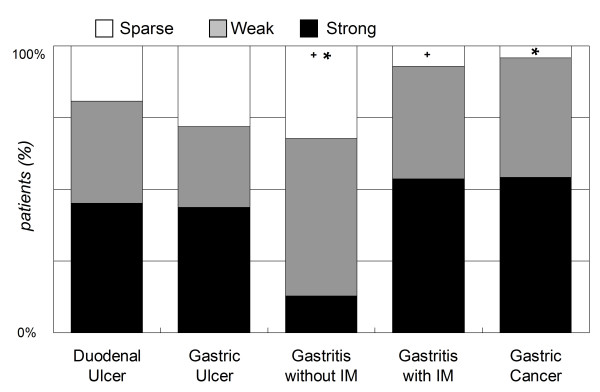
**The p-CagA intensity of the strains isolated from patients with different clinical categories**. The strains isolated from patients of gastric cancer or gastritis with intestinal metaplasia had stronger p-CagA intensity than those from gastritis without intestinal metaplasia patients (*p = 0.001, ^+^*p *= 0.002; Pearson chi-square test). IM = intestinal metaplasia.

**Figure 3:**
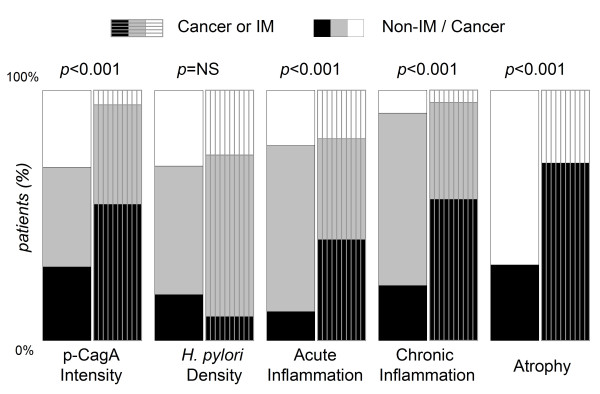
**Comparing with the isolates from patients without IM/cancer, those from cancer or IM patients had significantly stronger p-CagA intensity, more gastric atrophy, severer acute or chronic inflammation, but had no difference in *H. pylori *density**. (The black, grey & white bars indicate: strong, weak, & spare p-CagA; dense, moderate & loose *H. pylori *density; severe, moderate & mild inflammation; with & without atrophy.)

In Figure 2, the *H. pylori *strains of gastric cancer or gastritis with IM patients had stronger p-CagA intensity than those of gastritis without IM (54.2% & 53.6% vs. 12.9%, *p *≤ 0.002). There was also a trend that the *H. pylori *isolates from cancer or IM patients had relatively stronger p-CagA intensity then the subgroups of gastric and duodenal ulcer, but the difference was not significant. Moreover, the p-CagA intensity was not different among the subgroups of gastric ulcer, duodenal ulcer, and gastritis without IM. In Figure 3, the patients were separated according to having cancer risk or not. The isolates from the patients with cancer or IM had stronger p-CagA intensity than those from non-cancer/IM patients (p < 0.001). Furthermore, the patients with cancer risk had higher gastric inflammation or atrophy (p < 0.001).

The impacts of p-CagA intensity on gastric IM were analyzed in the non-cancer patients. Twenty-four out of the 47 patients (51.1%) infected with strong p-CagA strains had gastric IM. In contrast, for those with weak and sparse p-CagA, 35.4% (17 out of 48) and 11.1% (3 out of 27) patients had gastric IM. Accordingly, the patients infected with stronger p-CagA strains had higher prevalence of IM in the gastric specimens (*p *= 0.003). Moreover, the odds ratios of age, gender and p-CagA intensity on the gastric IM were showed in Table 2. As compared to those infected with strains with sparse p-CagA intensity, the crude odds ratio to have IM was 4.38 for those with weak p-CagA intensity, and increased to 8.34 for those with strong p-CagA intensity. Based on the logistic regression analysis to adjust the age, gender, and clinical diagnoses, the odds ratios to have IM were 3.93 for the patients infected with weak p-CagA intensity isolates and 10.45 for those with strong p-CagA intensity.

**Table 2:** The impacts of the p-CagA intensity of *H. pylori *on the gastric intestinal metaplasia in the 122 selected non-cancer patients by stratified analysis and logistical regression

	Odd ratio (95% CI)
**Crude**: Age < 50 years	1
< 50 years	8.14 (3.49~18.98)
Gender - Male	1
- Female	2.36 (1.12~5.11)
p-CagA - Sparse	1
- Weak	4.38 (1.15~16.72)
- Strong	8.34 (2.21~31.55)
**Age and gender adjusted**	
Sparse p-CagA	1
Weak p-CagA	3.67 (0.93~14.37)
Strong p-CagA	8.44 (2.08~34.12)
**Age, gender and disease adjusted**	
Sparse p-CagA	1
Weak p-CagA	3.93 (0.92~16.94)
Strong p-CagA	10.45 (2.25~48.48)

### Correlation between H. pylori p-CagA intensity and gastric histological features

**Figure 4:**
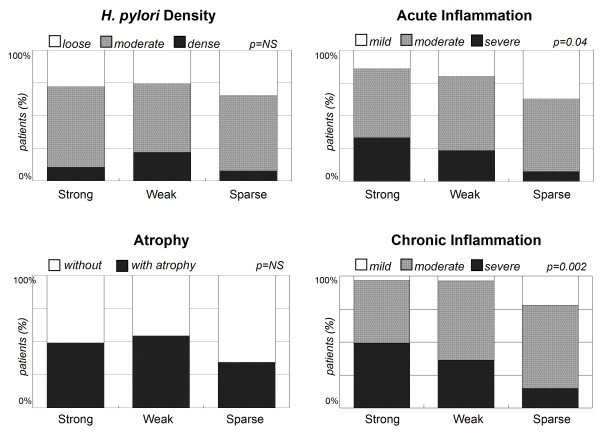
**The *H. pylori *density, inflammation and atrophy by gastric histology among the 146 patients infected with *H. pylori *isolates with different p-CagA intensity**. The isolates with stronger p-CagA intensity were significantly associated with more severe acute inflammation (*p *= 0.01) and chronic inflammation (*p *= 0.005) but not with *H. pylori *density or gastric atrophy (*p *= NS) (Pearson chi-square test).

In Figure 4, this study also analyzed whether there were an association between the p-CagA intensity and the severity of gastric inflammation in histology. The patients infected with *H. pylori *isolates with stronger p-CagA intensity may have more severe acute inflammation (*p *= 0.04) and also chronic inflammation (*p *= 0.002). Nevertheless, the p-CagA intensity of *H. pylori *isolates was not associated with the HPD or gastric atrophy (*p *> 0.05).

**Figure 5:**
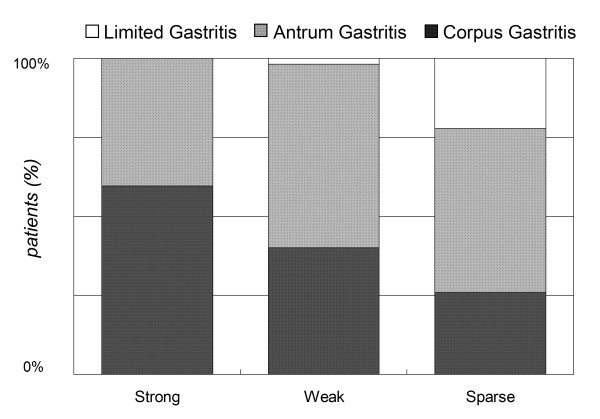
**The patients infected with strains with strong or weak p-CagA intensity had more corpus-predominant gastritis than those infected with strains with sparse p-CagA intensity (*p *= 0.001, Pearson chi-square test)**.

In Figure 5, a higher proportion of patients infected with a strain with strong p-CagA intensity had corpus-predominant gastritis (59.6%), as compared to those infected with *strains *with weak (40%) or sparse (25.9%) p-CagA intensity (*p *= 0.001). The adjusted odds ratio for age, gender, and clinical diagnoses by logistic regression was 3.15 (1.07~9.31) for patients infected with *H. pylori *with strong p-CagA intensity and 1.49 (0.51~4.35) for those infected with strains with weak p-CagA intensity, as compared with those with sparse p-CagA intensity.

## Discussion

This study shows the clinical impacts of *H. pylori *p-CagA intensity on the risk of gastric carcinogenesis. In Taiwan, the *H. pylori *isolates have universal presence of genes in *cag*-PAI and expression of CagA [13-16]. On the basis of the semi-quantitative analysis of the translocated p-CagA bands in the western blots, the strains in this study have diverse intensity of p-CagA. To further evaluate the clinical impact of the diverse p-CagA intensity on the clinical outcome, we selected a clinical strain with marked p-CagA to serve as reference index to subgroup the 146 collected strains according to their p-CagA intensity into strong, weak, or sparse. Based on this categorization, this study showed that *H. pylori *isolates with stronger p-CagA were correlated to more severe gastric inflammation and an increased risk of gastric IM and cancer.

The possible factors to affect CagA phosphrylation include the *cagA *genotype, type IV secretion system, the CagA EPIYA-repeat motif of the strain, and the adhesion phenotype of the epithelial cell [22-27]. Animal studies have shown that mutant strains of CagA, CagE, or CagY could reduce the gastric inflammation after infection [10,28]. Moreover, the CagA EPIYA polymorphism has also a causal role in clinical outcome [18,29]. These data support that these factors are all important in the *H. pylori *related gastric inflammation via CagA phosphorylation. However, there is no previous human study to evaluate the impact of the p-CagA intensity on gastric histological changes. Thus, this study is first time to disclose that strains isolated from gastric cancer and IM patients had a stronger p-CagA function as compared with strain from gastritis without IM patients (Figure 2). However, those were not significantly stronger than the strains from gastric or duodenal ulcer. This result can be explained that the IM and non-IM were both included into the gastric and duodenal ulcer subgroups to dilute the significance. This explanation may be also supported by a study showing that the intensities of p-CagA were not significantly different among different clinical diseases [22].

Moreover, as shown in Figure 3, the isolates from patients with cancer risk (i.e, patients with IM or cancer) had significantly stronger p-CagA intensity than those from patients without cancer risk (p < 0.001). This data further support that strong p-CagA increase the risk of developing gastric carcinogenesis from *H. pylori *infection. Furthermore, the patients with IM or cancer had severer acute and chronic inflammation in gastric histology. Also shown in Figure 4, the patients infected with stronger p-CagA *H. pylori *strains could correlate with severer acute or chronic gastritis (*p *< 0.05). This indicated that the p-CagA intensity is closely related to provoke gastric inflammation in both patients with and without gastric cancers.

It is well known that the *H. pylori*-infected host has an increased risk of developing gastric cancer, once the gastric histology reveals a corpus-predominant pattern or a precancerous change such as IM [30-33]. We, therefore, further validated whether the infection of patients with strong p-CagA *H. pylori *strains is associated with an increased risk of such histological changes. As shown in Figure 5, strains with stronger p-CagA caused more often corpus-predominant gastritis (*p *= 0.001). Also shown in Figure 2, the strains isolated from patients of gastritis with IM had a significantly stronger p-CagA than those from gastritis patients without IM (*p *= 0.002). These data supported the hypothesis that the p-CagA intensity of *H. pylori *isolates is closely related with the presence of IM.

In this study, instead of using all 469 stored strains, we systemically sampled 146 strains from our *H. pylori *database for the analysis of the p-CagA intensity. Both crude and adjusted odds ratio of the p-CagA intensity on IM were computed by logistical regression for the possible confounding factors, such as age, gender, and clinical disease. As shown in Table 2, the older age, female and stronger p-CagA had higher risk of having IM. In the multivariable regression, patients infected with *H. pylori *strains with strong and weak p-CagA had a 10.45 and 3.93 times higher risk of having IM than those infected with strains with sparse p-CagA intensity.

The study is noteworthy in showing that, in a 100% cagA-genopositive area, the p-CagA intensity could be an important independent factor closely associated with an increased risk of precancerous changes such as IM. However, the assessment of the p-CagA intensity in *H. pylori *isolates may not be widely available for clinical application. Accordingly, it is worth conducting future studies to determine biomarkers to indirectly evaluate the p-CagA intensity of the infected host. Once a biomarker is available, it will be helpful to identify patients infected with *H. pylori *strains with stronger p-CagA intensity, to determine the risk of gastric carcinogenesis in non-cancer patients, and then select these patients for earlier treatment.

## Conclusions

In conclusion, patients infected with a *H. pylori *strain with stronger CagA phosphorylation ability have more severe chronic gastric inflammation with an increased risk to have corpus-predominant gastritis, gastric intestinal metaplasia, and cancer.

## List of abbreviations used

p-CagA: phosphorylated-CagA; IM: intestinal metaplasia; cag-PAI: cag pathogenicity island; HPD: *H. pylori *density; AIS: acute inflammation score; CIS: chronic inflammation score; *H. pylori: Helicobacter pylori*

## Authors' contributions

**Guarantor of the article**: Bor-Shyang Sheu, MD

**Specific author contributions**: Dr. CCH and SBS initiated and coordinated the study conduction. CHC and CWL enrolled the patients. YHB reviewed the gastric histology. HKH, SSM, and WJJ assessed the *cagA *genotype and p-CagA intensity. All authors read and approved the final manuscript.

## Authors' information

Chiao-Hsiung Chuang, MD: Institute of Clinical Medicine, Department of Internal Medicine, Medical College, National Cheng Kung University, Tainan, Taiwan.

Hsiao-Bai Yang, MD: Department of Pathology, Medical College, National Cheng Kung University, Tainan; Department of Pathology, Ton-Yen General Hospital, Hsinchu, Taiwan.

Shew-Meei Sheu, PhD: Institute of Basic Medical Sciences, Medical College, National Cheng Kung University, Tainan, Taiwan.

Kuei-Hsiang Hung, PhD: Institute of Basic Medical Sciences, Medical College, National Cheng Kung University, Tainan, Taiwan.

Jiunn-Jong Wu, PhD: Institute of Basic Medical Sciences, Medical College, National Cheng Kung University, Tainan, Taiwan.

Hsiu-Chi Cheng, MD, PhD: Institute of Clinical Medicine, Department of Internal Medicine, Medical College, National Cheng Kung University, Tainan, Taiwan.

Wei-Lun Chang, MD: Institute of Clinical Medicine, Department of Internal Medicine, Medical College, National Cheng Kung University, Tainan, Taiwan.

Bor-Shyang Sheu, MD: Department of Internal Medicine, Institute of Clinical Medicine, Institute of Basic Medical Sciences, Medical College, National Cheng Kung University, Tainan, Taiwan.
